# Supplementary material for: The Rise of Fine-Tuned CAR-Based Therapies Against Acute Myeloid Leukemia
Source: Cancers (Basel). 2025 Dec 5;17(24):3892. doi: 10.3390/cancers17243892 (PMC12730820; doi:10.3390/cancers17243892)
Supplement: Supplementary file 1 [file cancers-17-03892-s001.zip › cancers-3968039-supplementary.pdf]

Supplementary Materials

Supplementary Table S1. Clinical trials evaluating emerging CAR-based cellular therapies in AML. Summary of target, phase, product type, data availability, and key findings for ongoing or recently completed early-phase trials.

Target	NCT Number	Phase	Product Type	Data Availability	Key Findings
CD7	NCT04538599	Phase I	Allogeneic fratricide-resistant CD7 CAR-T	[106]	ORR 81.8%, CR 63.6% in the treated cohort; only one AML patient was included, achieving MRD$^-$ CR. No DLTs, no ICANS, no GvHD, CRS ≤ 2. Limited persistence due to CD7$^-$CD8$^+$ T-cell allo-rejection; several responders proceeded to allo-HSCT.
CD7	NCT02742727	Phase I/II	NK-92 CD7 CAR-NK	No published data	-
CD19/CD20/CD22	NCT05418088	Phase I	Trispecific CD19/CD20/CD22 CAR-T	No published data	-
CD33	NCT05942599	Phase I	Allogeneic CD33 CAR-T	No published data	-
CD33	NCT04835519	Phase I	c-JUN overexpressing CD33 CAR-T	[113]	4 AML patients; CRS 1-2 except 1 CRS 4 DLT. Improved CAR persistence with c-JUN; 2 blast clearances + 1 partial response.
CD33	NCT03927261	Phase I/Ib	UltraCAR-T CD33-mbIL15	[72]	Successful 24h manufacturing; CAR expansion/persistence; acceptable safety; no response data reported.
CD33	NCT02944162	Phase I	NK-92 CD33 CAR-NK	No published data	-

CD33	NCT05665075	Phase I	Allogeneic CD33 CAR-NK	No published data	-
CD33	NCT05601466	Phase I	Allogeneic CD33 CAR-NK	No published data	-
CD33	NCT03795779	Phase I	Inducible/safety-switch CD33 CAR-T	No published data	-
CD33/CLL1	NCT05215015	Phase I	Dual-target CD33/CLL1 CAR-NK	No published data	-
CD33/CLL1	NCT05016063	Phase I	Dual CD33-CLL1 CAR-T	No published data	-
CD123	NCT03203369	Phase I	Allogeneic CD123 CAR-T	No published data	-
CD123	NCT03190278	Phase I	UCART123v1.2 (allogeneic CD123 CAR-T)	No published data	-
CD123	NCT02159495	Phase I	Suicide-switch/EGFRt CD123 CAR-T	No published data	-
CD123	NCT04318678	Phase I	CD123 CAR-T with CD20 safety switch	No published data	-
CD123 (UniCAR)	NCT04230265	Phase I	UniCAR02-T CD123 adapter CAR-T	No published data	-
CD123/CLL1	NCT03631576	Phase II/III (Phase I NCT03222674)	Dual CD123/CLL1 CAR-T	No published data	-
CLL1/CD123	NCT05654779	Phase I/II	Dual CLL1/CD123 CAR-T	No published data	-
CLEC12A	NCT06128044	Phase I	Autologous CLEC12A CAR-T	No published data	-

CLEC12A	NCT05748197	Phase I	Logic-gated synNotch CLEC12A CAR-T	No published data	-
CLEC12A	NCT06017258	Phase I	IL-18 TRUCK CLEC12A CAR-T	No published data	-
NKG2D	NCT04623944	Phase I	iPSC-derived NKG2D CAR-NK	No published data	-
NKG2D	NCT02944162	Phase I	NK-92 NKG2D CAR-NK	No published data	-
TIM-3	NCT05105152	Phase I	CAR-NK targeting TIM-3	No published data	-

ORR: overall response rate; **CR:** complete remission; **MRD:** minimal residual disease; **DLT:** dose-limiting toxicity; **CRS:** cytokine release syndrome; **ICANS:** immune effector cell-associated neurotoxicity syndrome; **GvHD:** graft-versus-host disease; **allo-HSCT:** allogeneic hematopoietic stem cell transplantation.
